# Case Report: Freeze-Dried Human Amniotic Membrane Allograft for the Treatment of Chronic Wounds: Results of a Multicentre Observational Study

**DOI:** 10.3389/fbioe.2021.649446

**Published:** 2021-06-24

**Authors:** Iveta Schmiedova, Zuzana Ozanova, Elen Stastna, Ludmila Kiselakova, Bretislav Lipovy, Serhiy Forostyak

**Affiliations:** ^1^PrimeCell Bioscience Inc., Ostrava, Czechia; ^2^BioHealing, Brno, Czechia; ^3^Department of Burns and Plastic Surgery, Faculty of Medicine, Institution Shared With University Hospital Brno, Masaryk University, Brno, Czechia

**Keywords:** biomaterials, amnion, clinical study, diabetes mellitus, wound management, biological dressing, regenerative medicine

## Abstract

An inability of the human body to heal acute wounds under certain conditions results in the formation of chronic ulcers. Chronic wounds not only cause significant pain and discomfort for patients but also serve as an entry for microorganisms into the human body, which can result in serious life-threatening problems and become a significant burden for the patients and society. The current work present results of a multicentre prospective observational study demonstrating the use of a lyophilized amniotic membrane (AM) in the treatment of chronic wounds (various etiologies). Lyophilized AM produced under the commercial brand Amnioderm® was used as an allograft material for therapy of chronic wounds, in addition to chronic ulcer standard-of-care (SoC) protocols. The duration of wounds considered for the application of AM ranged between 2 months and 11 years. In total, 16 patients were enrolled to the study, of which eight were completely healed, six demonstrated a significantly reduced ulcer size, and two did not respond to the AM therapy. The current study unambiguously demonstrates an effective alternative to the standard of chronic wound care and confirms a significant effect of the AM application for chronic wound management as a new SoC.

## Introduction

Debilitated wound healing is a major complication of diabetes type 1/2, with a lifetime risk of developing a diabetic foot ulcer (DFU) that is potentially as high as 25%. Total chronic wound incidence is expected to increase from 425 million in 2017 to 629 million in 2030 (Kathawala et al., 2019). Chronic wounds present a serious socioeconomic burden. The incremental cost of a DFU alone is US$20,000 annually in the United States with 1.5 million patients presenting with a new DFU each year; this costs $30 billion annually in the United States alone (Macdonald, 2009). The Wound Healing Society classifies chronic wounds into four categories: pressure ulcers (PUs), DFUs, venous ulcers (VUs), and arterial insufficiency ulcers (AIUs) (Kuehn, 2007).

Despite ongoing discussions about the time-related classification, the nomenclature generally defines a chronic wound as one that has failed to proceed through an orderly and timely reparative process to produce anatomic and functional integrity within 3 months (the time required for chronicity has been defined in the range of 4 weeks up to more than 3 months) or that has proceeded through the repair process without establishing sustained, anatomic, and functional results (Mekkes et al., 2003; Werdin et al., 2009; Jarbrink et al., 2016).

A common surgical approach aims at the promotion of epithelization by a combination of debridement manipulations (removal of non-vital tissues) and infection and inflammation management in combination with wound dressing with various coverings (antibiotics or silver covering). A recent comprehensive review on chronic wound management by Kathawala et al. (2019) selected four groups of wound healing technologies: biologics, biomaterials, cell-based technologies, and alternate innovations. Among those, the use of biomaterials derived from perinatal tissues is a very prospective approach, having several advantages in the treatment of patients with chronic wounds. These advantages are related to the unique mechanical, immunological, and regenerative properties of the amniotic membrane (AM). The human AM is the innermost, multi-layered part of the placenta (with a thickness of 0.02–0.5 mm), which contributes to the homeostasis of amniotic fluid during pregnancy. After birth, all perinatal tissues are considered biological waste, which in turn makes them easier to use for patients from an ethical perspective. AM properties have been widely described and clinically used. AM has been shown to promote epithelialization, reduce inflammation and fibrosis, promote neovascularization, and provide a substrate for cell growth and functions as a biological bandage (Sippel et al., 2001; Cirman et al., 2014). AM also contains some immunoregulatory factors, such as HLA-G and Fas ligand; well-documented re-epithelialization effects; and non-tumorigenic, antimicrobial, and anti-inflammatory properties (Koizumi et al., 2000; Kubo et al., 2001; Iranpour et al., 2018). AM also has been reported to have antibacterial and anti-inflammatory properties (John, 2003; Mao et al., 2017). In the wound healing process, AM contributes via the effect of cytokines and growth factors (GFs) which are present in fresh and frozen AM (Koob et al., 2015). Taken together, AM is a valuable tissue for the treatment of chronic wounds and for regenerative medicine.

The objective of the current clinical research was to compare healing characteristics (wound reduction and rates of complete wound closure) of the new product Amnioderm®, based on an AM, when included in the standard chronic wound care in patients with incurable chronic foot ulcers. Amnioderm® is a freeze-dehydrated human AM manufactured by the patented technology Amnipur®, which preserves healing properties of the AM for at least 5 years (shelf-life) at room temperature. It is one of the first multicentre randomized prospective observational case studies conducted in Central and Eastern Europe, evaluating the use of an AM in diabetic and non-diabetic patients with chronic wounds. An application of AM has been performed in the following centers: Levoča (General Hospital Levoča, a.s.), Lubochňa (National Endocrinology and Dialectology Institute), Jihlava (Jihlava Hospital), Sokolov (Sokolov Hospital), Svidník (DOST), Tňinec (Salvatella, s.r.o.), and Brno (Faculty Hospital Brno).

## Methods

### Amniotic Membrane Collection and Manufacturing

AM was collected from the donors who have signed informed consent for donation of perinatal tissues for allogeneic use. All procedures and manipulations were performed following the ethical standards of the institutional and/or national research committee and in accordance with the 1964 Helsinki Declaration and its later amendments or comparable ethical standards. Perinatal tissues and blood samples have been collected from donors after the cesarean cut (CS). Blood serums of all donors considered for perinatal tissue donation were tested following the guidelines of valid Czech national legislation. Suitable donors were negatively tested for all of the following parameters: HIV 1/2 Ag/Ab, HBsAg, HBc total Ab, HCV Ab, HTLV 1/2 Ab, and *Treponema pallidum* Ab. The results of the above tests were negative for the presence of any infectious agents. All tests were performed in the diagnostic laboratories authorized by the Czech State Institute for Drug Control (SIDC). Following collection and transportation to the manufacturing clean facility, AM was separated from the placenta and purified (patented technology Amnipur®, patent no. 307603). This technology allows preservation of important biologically active ingredients and storage of AM at room temperature, enabling the possibility of immediate application. The characteristics of the Amnioderm® structure and its composition were determined using liquid chromatography–mass spectrometry analysis and additionally scanning electron microscopy (Supplementary Figure 1).

### Patients' Enrolment and Study Design

A multicentre, prospective, randomized, comparative, observation, non-blinded case study, without a placebo control group, to assess the healing effect of a freeze-dehydrated human AM (Amnioderm®, BioHealing, http://biohealingeurope.eu/en/home-page/) has been conducted following the ethical guidelines of the 1975 Declaration of Helsinki. Patients read and signed an informed consent form (ICF) before inclusion to the study. Amnioderm® is classified as a tissue transplant. The current study enrolled seven specialized centers focused on chronic wound therapy and management (the Czech Republic and the Slovak Republic). The study enrolled 16 patients suffering from chronic ulcers of diabetic (diabetes mellitus type II, DM) and of non-diabetic origin (NDM). Subjects were enrolled in the study following the inclusion/exclusion criteria described in Table 1 and following the same therapeutic protocol.

**Table 1:** Major inclusion and exclusion criteria.

**Inclusion criteria**	**Exclusion criteria**
• The patient's age—older than 18 years • The duration of inefficient chronic wound therapy—longer than 2 months (the upper limit of the wound age has not been established) • No clinical signs of infections • The size of the treated defect is in the sum of up to 16 cm^2^ • Good vascular patency without clinical symptoms of macroangiopathy. Patients with ischaemic artery disease of the lower extremities after successful revascularization • The patient shall sign their informed consent with the application of an amniotic membrane—Amnioderm®	• Active participation in another ongoing clinical study • Active radiotherapy or chemotherapy • Pregnancy or breastfeeding • Patient with anamnesis of oncological disease • Use of medications considered to be immune system modulators • Allergy or known sensitivity to gentamicin or gentian violet • Patient with polyvalent allergy and skin manifestation of unclear etiology

### Management of the Patients and Amnioderm® Application

The basic principles of chronic wound care are based on the concept of wound management and care, which primarily requires the determination of cause and identification of factors that could delay healing. Another prerequisite of successful healing is proper debridement, management of infection, and inhibition of inflammation, exudate, and swelling as well as establishment of an optimal moist environment in which cellular and biochemical procedures of the healing process can occur. Another therapeutic step is the promotion of epithelization. It is emphasized that care must be complex with an approach differentiated to the patient (Saap et al., 2004; Kuehn, 2007).

Patients enrolled in the study were questioned on whether they were a smoker/non-smoker and were tested for their body mass index (for details, see **Table 4**). All patients were managed following the standard clinical protocol of chronic wound management (schematic presentation in Table 2). During the first visit to the center, patients enrolled to the study underwent mechanical debridement of the wound (the wound must not show signs of infection) and application of antiseptic, followed by cleaning with normal saline solution. The initial size of the wound has been photographed and measured using a standard scale ruler (see Figure 2). Baseline and dynamic wound sizes have been evaluated to follow the dynamics of the healing process. Amnioderm® has been applied by clinicians in addition to the standard of care (SoC). DM (type II) patients enrolled to the study were tested for blood glucose level and the level of glycosylated hemoglobin. The size of the AM used for the application directly to the wound was slightly larger than the actual wound area so that it would extend beyond the edges. Hydration of the AM (primary covering) was performed with normal saline solution (0.9% NaCl in water) before being covered with a non-adhesive silicone dressing (secondary dressing). Within 1 week, Amnioderm® tightly adheres to the wound, thus serving as dressing material, which promotes the healing processes, prevents microbial contamination, and reduces pain/pruritus. The second and every subsequent procedure of Amnioderm® application were similar to the first one. The overall number of AM applications depends on the response to the therapy and varied between the patients.

**Table 2:** Wound management strategy (AD, Amnioderm®).

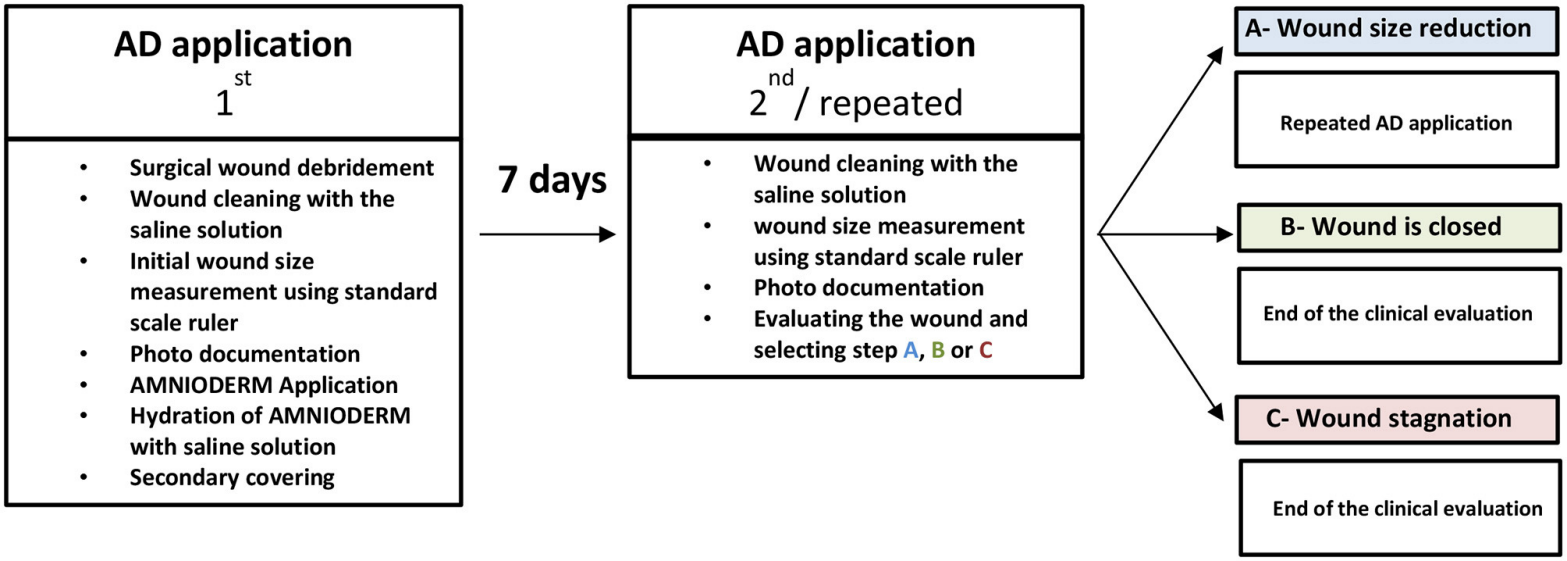

### Clinical Data Collection and Statistical Analysis

Complete wound closure has been identified when at least 95% of the initial wound size has been closed. Wound closure within the 95–50% range of the initial size has been identified as uncomplete. Wounds which had a closure smaller than 50% of the initial size has been identified as non-responders. The study has established a limit of a maximum of 14 applications of AM in addition to the SoC, which corresponded to the 14 weeks of patient observation. When the wound has been closed or AM application limit has been reached, data have been collected and analyzed by independent researchers who were not in direct contact with the patients. The following criteria have been evaluated (Table 3):

- Gender.
- Localization.
- Etiology.
- Patient's age.
- Wound's age.

**Table 3:** Patients characteristics^*^.

**Gender**	
Male	6 (37.5%)
Female	10 (62.5%)
**Wound localization**	
Left extremity	9 (56.3%)
Right extremity	7 (43.7%)
**Wound etiology**	
Diabetes mellitus (DM)	10 (62.5%)
Chronic venous insufficiency (NDM)	4 (25%)
Wound after abscess (NDM)	1 (6.25%)
An abscess (NDM)	
Other (NDM)	1 (6.25%)
**Age (years)**	**66.0 (48.9–81.6)**
<50	1 (6.3%)
50–59	3 (18.8%)
60–69	7 (43.8%)
70–79	3 (18.8%)
≥80	2 (12.5%)
**Median age (years), DM group**	64.0
**Median age (years), NDM group**	71.0
**Wound duration (months)**	**18.0 (4.8–132.0)**
<6	1 (6.3%)
6–11	3 (18.8%)
12–17	2 (12.5%)
18–23	4 (25.0%)
24–35	2 (12.5%)
≥36	4 (25.0%)
**Median duration of the wound (months), DM group**	24.0
**Median duration of the wound (months), NDM group**	6.0
**Baseline wound size (cm**^**2**^**)**	**1.2 (0.4–7.5)**
<1.0	6 (37.5%)
1–5.9	8 (50.0%)
≥6.0	2 (12.5%)
**Median baseline wound size, DM (cm**^**2**^**)**	1.4
**Median baseline wound size, NDM (cm**^**2**^**)**	2.1

*DM, diabetes mellitus; NDM, non-diabetic origin*.

* *Data presented as mean, median (min, max), or number (%)*.

To follow the dynamics of wound closure, we have processed images with the ImageJ software [National Institutes of Health and the Laboratory for Optical and Computational Instrumentation (LOCI), University of Wisconsin] and measured the area of the wound over time. Calibration of the software has been performed using a standard scale used during the photo documentation of the wound size. We have evaluated the following criteria:

- The efficacy of AM therapy in all patients.
- The efficacy of AM treatment in the DM vs. NDM groups.
- The dynamics of wound healing during the whole course of the therapy (Kaplan–Meier wound closure plot).

Other followed subjective criteria such as pain and itching were tested using a standard questionnaire.

## Results

### Patient Enrolment

The study comprised representative types of patients typically seen in the community. Eligible patients were divided into two groups: type II DM (*N* = 10) and non-diabetic etiology of chronic wounds (NDM, *N* = 6). Main inclusion criteria were determined by a history of patients receiving treatment for a chronic ulcer of at least 8 weeks' duration. All eligible patients were offered enrolment as long as they met the approved study inclusion and exclusion criteria described above. Detailed characteristics of enrolled patients are described in Tables 3, 4. Sixteen subjects were enrolled and randomly assigned to the study between January and July of 2018. The male-to-female ratio of the patients in the study was: 62.5% male to 37.5% female. The median age of the patients involved in the study was 66 years, while 93.7% of the patients were older than 50 years. Out of 16 observed subjects, seven (43.7%) were localized on the right lower extremity and nine (56.3%) on the left lower extremity. We enrolled 10 patients (62.5%) with a confirmed diagnosis of DFU, whereas six wounds (25.0%) were related to chronic vein insufficiency [venous leg ulcer (VLU)]. Median duration of inefficient chronic wound management among all subjects was as follows: <6 months (6.3%, one subject); 6–11 months (18.8%, three subjects); 12–17 months (12.5%, two subjects); 18–23 months (25.0%, four subjects), 24–35 months (12.5%, two subjects); and 36 months (25.0%, four subjects). Baseline wound size were as follows: <1 cm^2^ (six patients, 37.5%); 1–5 cm^2^ (eight patients, 50.0%); and more than 6 cm^2^ (two patients, 12.5%).

**Table 4:** Characteristics of the patients enrolled in the study: smoker and body mass index (BMI).

**Patient ID**	**Smoker**	**BMI**	**SEX**	**Chronic wound duration**	**Duration of treatment/number of AM application (weeks)**	**DM/NDM**	**Result (healed/partially healed/non-responder)**
1	N	40.69	M	18	7	DM (II)	Partially healed
2	N	31.58	F	36	17	DM (II)	Partially healed
3	N	29.37	M	24	6	DM (II)	Healed
4	N	26.79	M	18	16	DM (II)	Partially healed
5	N	30.16	M	60	17	DM (II)	Non-responder
6	N	38.61	M	24	17	DM (II)	Non-responder
7	N	31.89	F	132	9	DM (II)	Healed
8	N	31.89	F	132	9	DM (II)	Healed
9	N	34.89	F	18	10	DM (II)	Healed
10	Y	25.59	F	12	13	NDM	Healed
11	Y	30.6	M	18	6	DM (II)	Partially healed
12	N	29.74	F	2	7	NDM	Healed
13	N	18.6	F	6	9	NDM	Healed
14	N	19.2	F	6	10	NDM	Partially healed
15	N	28.2	F	12	4	NDM	Partially healed
16	N	29.05	F	6	9	NDM	Healed

The study aimed to evaluate NDM origin as an alternative wound care treatment protocol to the standard chronic wound management. All subjects were monitored for objective and subjective parameters established for the groups before the first application. The study protocol was designed for 16 weeks. All enrolled subjects had the option of exiting the study either due to the complete wound healing or the failure to achieve at least a 50% reduction in ulcer size after 16 weeks of study enrolment.

**Figure 1:**
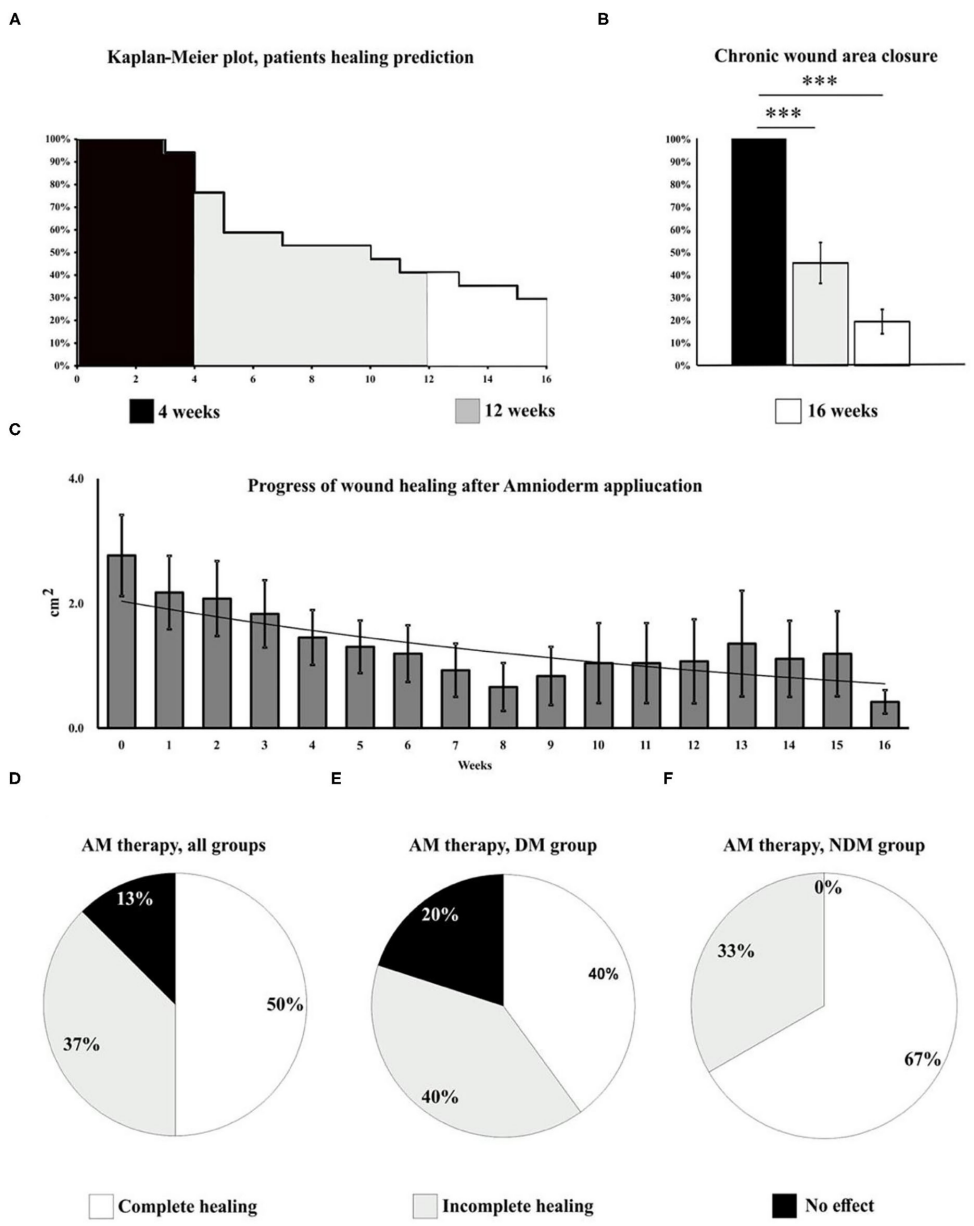
Wound healing progress has been evaluated in all subjects included in the study for 16 weeks. The Kaplan–Meier plot shows the dynamics of patients exiting the study and predicts the healing potential of AM over time **(A)**. During the first 4 weeks of the study, wound granulation and epithelization were observed in 43.8% of the patients (*N* = 7), three of which exited the study due to the complete wound healing. Between weeks 3 and 12 **(A)**, 80% wound size reduction **(B)** and total healing in 50% of all subjects (*N* = 8) enrolled to the study **(C**, **D)** have been observed. The study shows complete ulcer healing in half of the patients, 37% of the wounds had a significant size reduction (up to 90% of baseline size), and 13% of the ulcers were not responding to the therapy (*N* = 2). Therapy outcomes, DM group **(E)**: complete healing (40%, *N* = 4), significant reduction of the ulcer size (40%, *N* = 4), and unresponsive to the therapy (30%, *N* = 2). Therapy outcomes, NDM group **(F)**: complete healing (66%, *N* = 4), significant (up to 83%) reduction of the ulcer size (33%, *N* = 2), and unresponsive to the therapy (0%). A very significant changes (*p* < 0.001) are marked as***.

**Figure 2:**
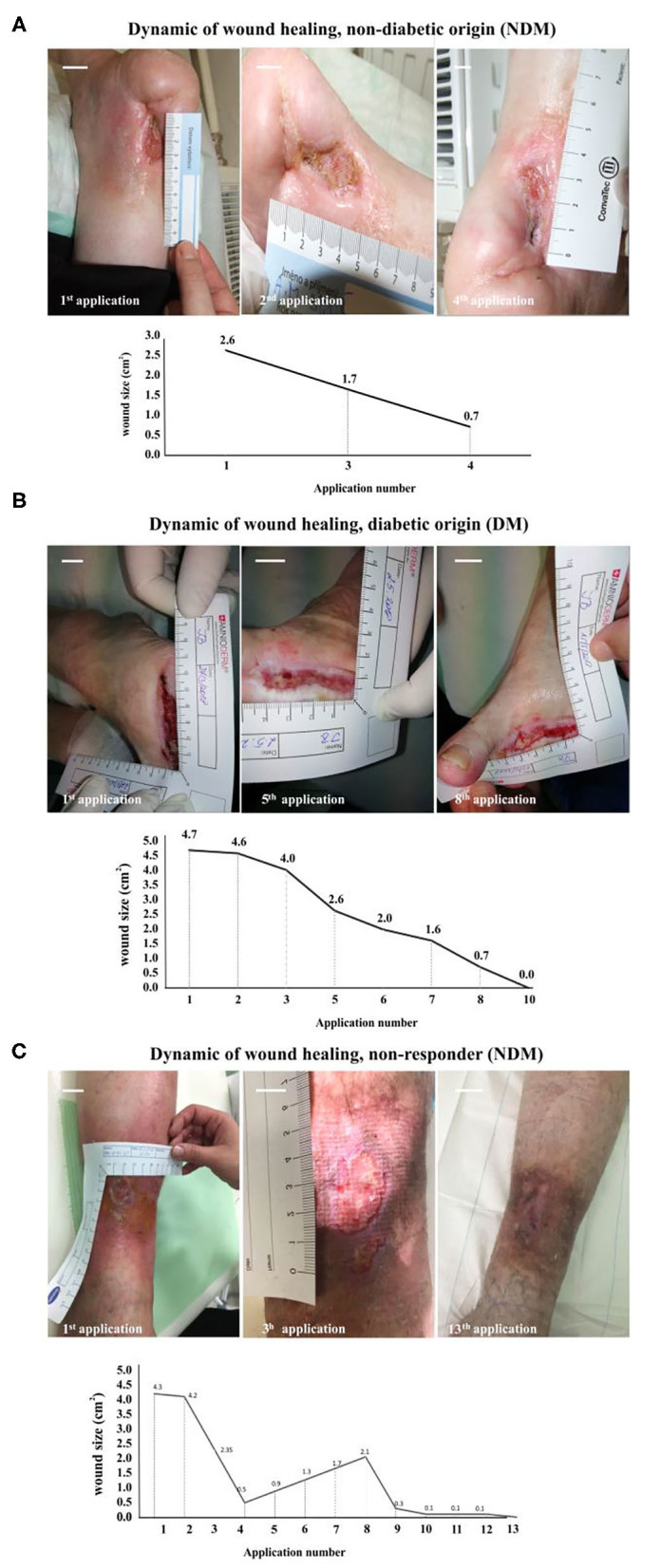
Wound healing dynamics after Amnioderm® application: NDM **(A)**, DM **(B)**, and non-responder NDM **(C)** groups. A female patient (69 years old) suffering from a non-diabetic chronic wound for 12 months **(A)**. After four Amnioderm® applications, a significant reduction of ulcer size was observed, leading to complete healing after 6 weeks. An example of a female patient (58 years old) with a confirmed diagnosis of DM, suffering from a chronic ulcer at the lower limb for 18 months **(B)**. After 4 weeks of Amnioderm® therapy, the wound completely healed by the 10th week. An example of a patient (female, 67 years old) with a history of chronic wound of venous etiology **(C)**. Applications of AM led to a significant reduction of pain and size of a wound, but not complete healing. Scale bars = 1 cm. NDM, non-diabetic patients; DM, diabetes mellitus type II patients.

We identified two major groups of patients: responders (87%) and non-responders (13%). Among the responders group, 50% of patients had a completely healed ulcer, while the other 37% had a significantly (up to 90% from the baseline size) reduced ulcer size. An active ulcer regeneration process has been observed between weeks 3 and 12 (Figure 1) when more than 80% wound size reduction (Figures 1B,C) and total ulcers healing in more than 50% of all subjects enrolled to the study were observed (Figures 1C,D). During the first 4 weeks of AM therapy, wound granulation and even epithelization were observed in 43.8% of the patients (*N* = 7), and two patients were healed (defined as complete epithelialization of the open area of the wound) within 4 weeks of study enrolment. An overall evaluation of all subjects in the study shows that 50% of all ulcers were completely closed, 37% of the treated wounds had a significant ulcer size reduction (39–99%), and 13% of the wounds were not responding to the therapy (*N* = 2). Patients with DM (*N* = 10) had total wound closure in 40% of cases (*N* = 4), and partial healing (39–99%) was observed in 40% of cases (*N* = 4), while 20% of DM ulcers (*N* = 2) were unresponsive to the therapy (Figure 1E). Non-diabetic ulcers have been healed in 67% of the group (*N* = 4), while the other 33% of the subjects (*N* = 2) had a significant decrease of the chronic wound size up to 83% from the baseline wound size (Figure 1F). The average ulcer size in patients at the beginning of Amnioderm® application was 2.8 cm^2^ and gradually decreased after subsequent applications (second to sixteenth) (Figures 1B,C). From week 12 of AM application, four patients were observed, of which two had a significant reduction of ulcer size (up to 90% from the baseline) and two patients exited the study with the same wound size as before the first AM application (DM group). While before the first application, 34.6% of subjects reported pain in the wound area, when exiting the study, all patients (100%) had no painful feeling in the area of the ulcers. Similar to the pain, 34.6% of subjects reported pruritus in the chronic wound area before the AM application, while there was no reported feeling of scratching during the exit from the study. During the study period, none of the patients experienced adverse events. The wound-healing dynamics after AM application is shown in Figure 2: NDM (A), DM (B), and non-responder NDM (C) groups.

## Discussion

The use of human AM was first described in 1910. Davis (1909) reported several cases of transplantations to treat acute and chronic wounds. Among the skin grafts used, AM also appeared as a biological dressing; however, it was a non-processed form. In the following period, publications gradually began to appear, pointing to the possibility of using conserved AM in various types of wound (Sabella, 1913; Stern, 1913). In 1940, the pro-healing effect of AM was also used in the case of ocular (conjunctival) lesion management—AM and chorion were used in this indication (de Rotth, 1940). Since then, there has been a significant acceleration in the use of AM either alone or in combination with chorion in several indications such as burns, PUs, and DFUs as well as in the case of rare skin disorders (toxic epidermal necrolysis or epidermolysis bullosa) (Lo et al., 2010; Klama-Baryla et al., 2020).

One of the most common forms of AM preservation is dehydration [dehydrated human amniochorion membrane (dHACM)]. Pioneering information on the systematic use of dHACM in various chronic wounds was reported in 1950 (Troensegaard-Hansen, 1950). According to the available information, the superiority of this biological dressing has been demonstrated in chronic wounds with different etiologies (Niknejad et al., 2008).

Although we know that AM has many very-well-defined biological activities, the number of processes that can be modulated by AM is still unknown. The basic precondition for the success of the use of AM in non-healing chronic wounds is the reversal of the whole process and the optimization of the wound microenvironment by supplying a wide range of GFs [vascular endothelial growth factor (VEGF), platelet-derived growth factor (PDGF)-AA, PDGF-BB, transforming growth factor (TGF)α, TGFβ1, epidermal growth factor (EGF), basic fibroblast growth factor (bFGF)] and cytokines [interleukin (IL)-4, IL-6, IL-8, and IL-10] (Grzywocz et al., 2014; Koob et al., 2014a; Mcquilling et al., 2017). According to many authors, in addition to VEGF in AM, there are also several other proangiogenic cytokines (angiogenin, angiopoietin-2, etc.), which have the potential to increase neovascularization in the wound area and optimize transcutaneous oxygen pressure (tcpO_2_), which has a clear impact on wound healing, especially in ischaemic skin defects (Koob et al., 2014b; Wang et al., 2016). Nevertheless, the biological activity of AM can be relatively well-defined mainly due to the composition of their cytokines. Koob et al. (2015) tried to define the representation of individual GFs and cytokines in the amnion and chorion regions, using a commercial product called PURION® for this purpose. Interestingly, the amounts of the individual biologically active components were virtually identical in both the amnion and the chorion. According to their measurements, there are five dominant GFs: tissue inhibitor of metalloproteinase (TIMP)-4 (amnion: 5,993 pg/mg and chorion: 5,958 pg/mg dry mass of tissue), bFGF (4,455 and 4,276 pg/mg), TGFα (3,208 and 4,216 pg/mg), PDGF-AA (2,151 and 4,565 pg/mg), and TIMP-2 (228 and 378 pg/mg), which were also discovered in Amnioderm®.

In the context of wound healing, the issues of the frequency of dressings and the replication of AM itself are also widely discussed. Most chronic wound healing studies apply AM one to two times per week as part of their recommendation (Smiell et al., 2015). The frequency of dressings can be very intensely influenced by several internal but also external factors: type of wounds (degree of water discharge), infection (bioburden presence), and protease activity, phase of wound healing (granulation and epithelization), patient compliance, and others.

This is the first multicentre observational clinical study in Central and Eastern European counties (Czech Republic and the Slovak Republic) and to our knowledge in the EU, evaluating the effect of standard chronic wounds care in combination with a new biological material—a dehydrated human AM in patients with long-standing diabetic and non-diabetic chronic ulcers. Patients treated with Amnioderm® had significantly greater rates of regeneration and wound closure when compared to the earlier history of ineffective SoC for a substantial period (up to 12 months). Amnioderm® has been shown to facilitate regeneration of chronic non-healing ulcers of diabetic and non-diabetic origins in 87% of all cases with complete wound closure in 50% of enrolled subjects.

Positive effects of the perinatal tissues have been previously demonstrated in several studies where amnion and/or amniochorion membrane-based products were used (Faulk et al., 1980; Forbes and Fetterolf, 2012; Zelen et al., 2013; Castellanos et al., 2017). We have summarized the outcomes of the studies in Supplementary Table 1 (Zelen, 2013; Sheikh et al., 2014; Zelen et al., 2014, 2016; Mrugala et al., 2016). All of the above studies enrolled patients with chronic wound history between 2 weeks and 12 months before the first AM product application in addition to the SoC (surgical debridement plus wound management). The current study applied a similar strategy of wound management in addition to the SoC. We demonstrate the beneficial effect of Amnioderm® application in chronic wound cases of DM and NDM origins. Zelen et al. (2013) compared the efficacy of dHACM in chronic wound patients randomized to two groups: the first group had SoC alone, and the second group had SoC with the use of dHACM application two times per week. At 4 weeks after the first application, it was already possible to observe significant changes in the level of wound reduction between the two groups (32.0% ± 43.7% SoC, *N* = 12 vs. 97.1% ± 7.0% dHACM, *N* = 13). Similar results were obtained by Serena et al., which compared the effectiveness of dHACM EpiFix® + multilayer compression vs. SoC (multilayer compression alone). After 4 weeks, more than a 40% reduction in wound size was seen in 62% of patients in the treatment arm and in only 32% in the SoC arm (*p* = 0.005). The same biomaterial was also studied by Bianchi et al. (2018) on a total of 109 subjects. Weekly application of dHACM in the treatment arm led to a significantly higher probability of wound closure in both monitored periods (60% in the treatment arm vs. 35% in the SoC arm after 12 weeks, *p* = 0.0127 and 71% vs. 44% after 16 weeks, *p* = 0.0065). In 2019, Tettelbach et al. (2019) showed the results of their multicentre study on a total of 110 patients with DFUs. Of these patients, a total of 98 were treated according to treatment protocol (47 patients with dHACM, 51 patients with non-dHACM). The primary outcome of this study was to determine the percentage of patients with complete wound closure 12 weeks after the first application. After this time, it was found that 81% of patients with dHACM had a completely closed skin defect, while within the SoC, it was only 55% (*p* = 0.0093).

Anticipated results exceeded expectations compared with earlier studies. An average wound healing period to reach either full healing or significant decrease (87%) of ulcer size was 8 weeks in subjects, with the duration of chronic ulcers persisting between 6 months and 13 years. This could be explained by a manufacturing process preserving the rich composition of bioactive substances in AM tissues. Liquid chromatography–mass spectrometry analysis revealed more than a thousand active compounds in Amnioderm®. We have grouped these components into biological groups which are actively involved in the wound healing process as follows: protease inhibitors, cytokines, enzymes, extracellular matrix proteins, GFs, hormones, matrix metalloproteinases, and neurotrophic factors. Earlier, we reported a facilitated scarless regeneration of the skin after Amnioderm® application on the thermal wounds (Lipovy and Forostyak, 2020). AM contains extracellular matrix proteins including hyaluronic acid, which inhibits excessive fibrotization and reduces scarring and undesired accretions in the wound which also promotes chronic wound healing (Mohammadi et al., 2017; Kennedy et al., 2018). A cocktail of GFs [EGF, keratinocyte growth factor (KGF), hepatocyte growth factor (HGF), bFGF, fibroblast growth factor (FGF), and TGFβ], major anti-inflammatory ILs (e.g., IL-10), and thrombospondin-1 (which are antagonists of the IL-1 receptor and TIMPs) supports and activates migration, proliferation, and differentiation of epithelial cells and significantly supports epithelization, new capillary formation (neoangiogenic effect), and wound regeneration (Insausti et al., 2010; Koob et al., 2014b; Elheneidy et al., 2016). Additionally, several reports explain the decrease of pain and itching after AM application by the covering of the loose nerve twigs in the wound, which reduces the anti-inflammatory cytokines and peptides and significantly reduces pain (Diaz-Prado et al., 2011; Mohan et al., 2017).

Another aspect to look at in the outcomes of the study is economics. Considering clinical and operational efficiency for the clinicians and improvement of patients' quality of life along with cost-effectiveness, the use of Amnioderm® shall be seen as a therapy of choice. While healthcare economists could claim that use of modern biological material for wound management are tangible costs, but accurate economic analyses shall consider and develop from productivity and quality of subjects' life. The limitations of this study are those inherent to a small sample size. We have identified a group of responders and non-responders to AM therapy, and therefore a larger clinical data collection is needed. We are currently underway to addressing these questions. Additional comparative effectiveness studies with different materials and bigger groups of chronic wounds of various origin would be beneficial to specify more accurate indications and timing for Amnioderm® application. As part of the use of AM or AM + chorion in patients with DFUs, two meta-analyses were also published that summarized efficacy and safety (Su et al., 2020). In Huang et al., a total of nine randomized controlled trials (RCTs) with a total of 541 patients are included. Compared to SoC, the treatment arm + SoC improved DFU healing rates at 6 weeks (RR = 3.50, 95% CI: 2.35–5.21), 12 weeks (RR = 2.09, 95% CI: 1.53–2.85), and 16 weeks (RR = 1.70, 95% CI: 1.25–2.30). Several materials containing either HAM alone or in combination with the chorion, with different processing methodologies (dehydration, cryopreservation, decellularization, or hypothermic preservation), were used in the studies. The average cost of application of dHACM was $2,798 (SD: $4,528) in the Zelen et al. (2016) study and $1,771 (SD: $1,375) in the (Didomenico et al., 2018) study, and the median cost was $2,252 in the study of Tettelbach et al. (2019). The problem of all current studies on a given topic is also the minimal information regarding the quality of subsequent life. More studies will have to be carried out in this aspect; otherwise, the higher costs of using this biomaterial will be difficult to discuss and evaluate.

In conclusion, the application of dehydrated human AM demonstrated superior clinical effectiveness, when compared with the outcomes of SoC for chronic diabetic and/or non-diabetic ulcers at the lower extremities, applied to the subjects before the enrolment to the current study. Amnioderm® has excellent handling characteristics and operational efficiency, is ready to use, can be transported and stored at room temperature for up to 5 years, has minimum need for complex policies for receiving and storing the material, comes in different sizes, and thus can be used on varying ulcer sizes and at different stages of wound healing. The above features minimize the time for application and minimize the amount of waste, allowing more efficient utilization of the clinicians' time and efforts. Therefore, it appears to be a clinical and economical option to be implemented as a standard of chronic wound care in diabetic and non-diabetic patients.

## Data Availability Statement

The raw data supporting the conclusions of this article will be made available by the authors, without undue reservation.

## Ethics Statement

Ethical review and approval was not required for the study on human participants in accordance with the local legislation and institutional requirements. The patients/participants provided their written informed consent to participate in this study.

## Author Contributions

IS, ZO, ES, and LK contributed to the data collection, processing and draft writing. IS, ES, and SF conceptualization, data analysis. IS, BL, and SF writing of the final manuscript version. All authors contributed to the manuscript revision, read, and approved the submitted version.

## Conflict of Interest

IS, ZO, ES, LK, and SF were employed by PrimeCell Bioscience Inc. IS, ZO, ES, LK, and SF were employed by Biohealing Inc. The remaining author declares that the research was conducted in the absence of any commercial or financial relationships that could be construed as a potential conflict of interest.

## Supplementary Material

Supplementary Figure 1 Results of a liquid chromatography–mass spectrometry analysis of Amnioderm® revealed traces of 318 proteins. The proteins have been clustered into three major groups enrolled to wound healing: enzymes **(A)**, extracellular matrix proteins **(B)**, and cytokines **(C)**. Relative quantities of the protein contents have been also color-coded within each group (yellow: highest content and blue: lowest); protein relative numbers below 20 have not been included in the analysis (cut-off). A microscopic structure of the Amnioderm® has been studied by scanning electron microscopy, which has revealed preservation of its native structure: basement membrane (smooth structure) and intermediate (spongy) layer. Scale bar = 2 μm.

Supplementary Table 1 Comparison of the Amnioderm therapy outcomes with similar studies using AM for chronic wound therapy. dHAM, dehydrated human amniotic membrane; dHACM, dehydrated human amniochorion membrane; NA, non-applicable.
