# Supplementary figures and images for: Case Report: Freeze-Dried Human Amniotic Membrane Allograft for the Treatment of Chronic Wounds: Results of a Multicentre Observational Study
Source: Front Bioeng Biotechnol. 2021 Jun 24;9:649446. doi: 10.3389/fbioe.2021.649446 (PMC8264202; doi:10.3389/fbioe.2021.649446)

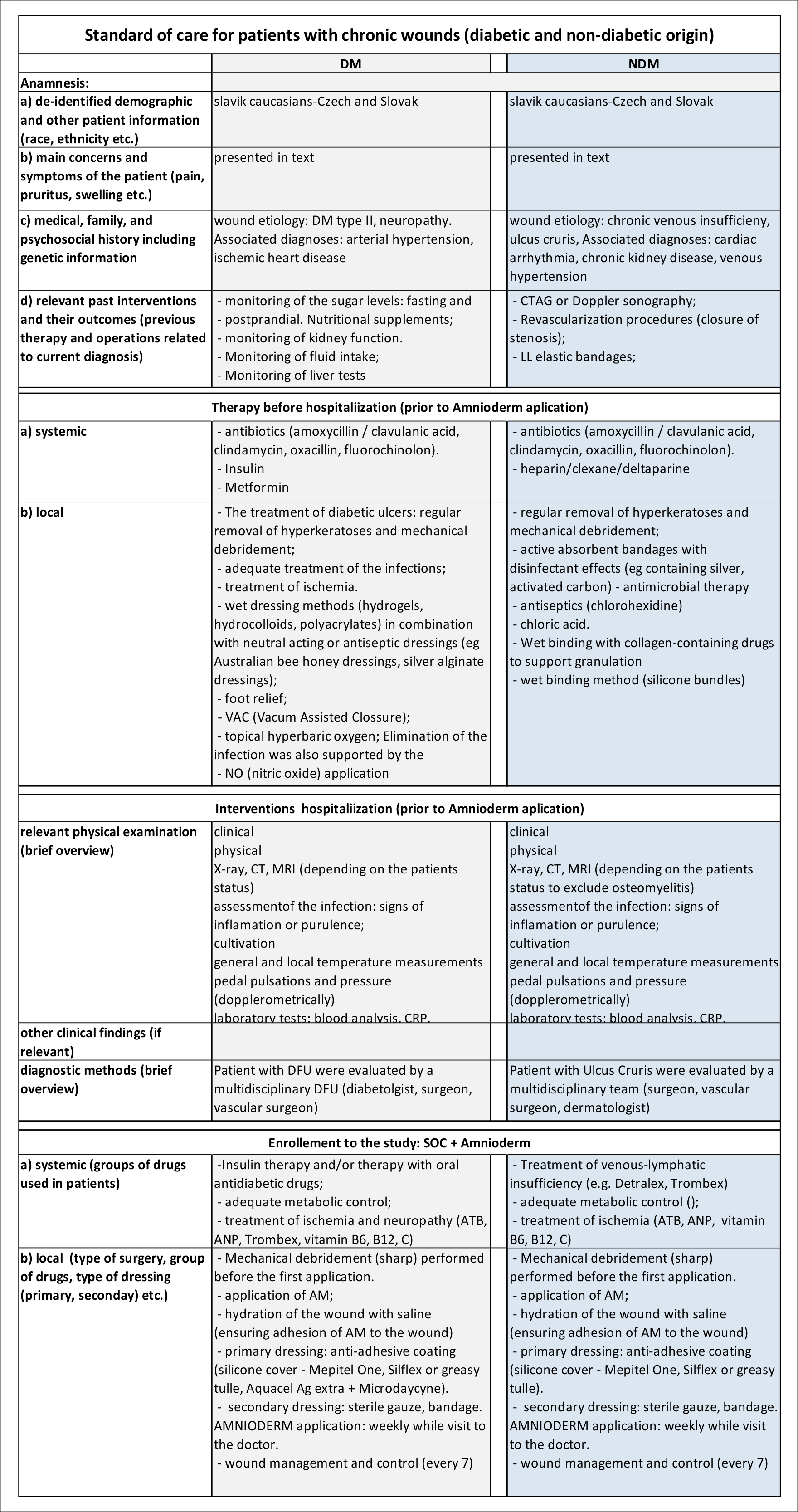

Supplement: Supplementary file 1 [file Image_2.TIF]

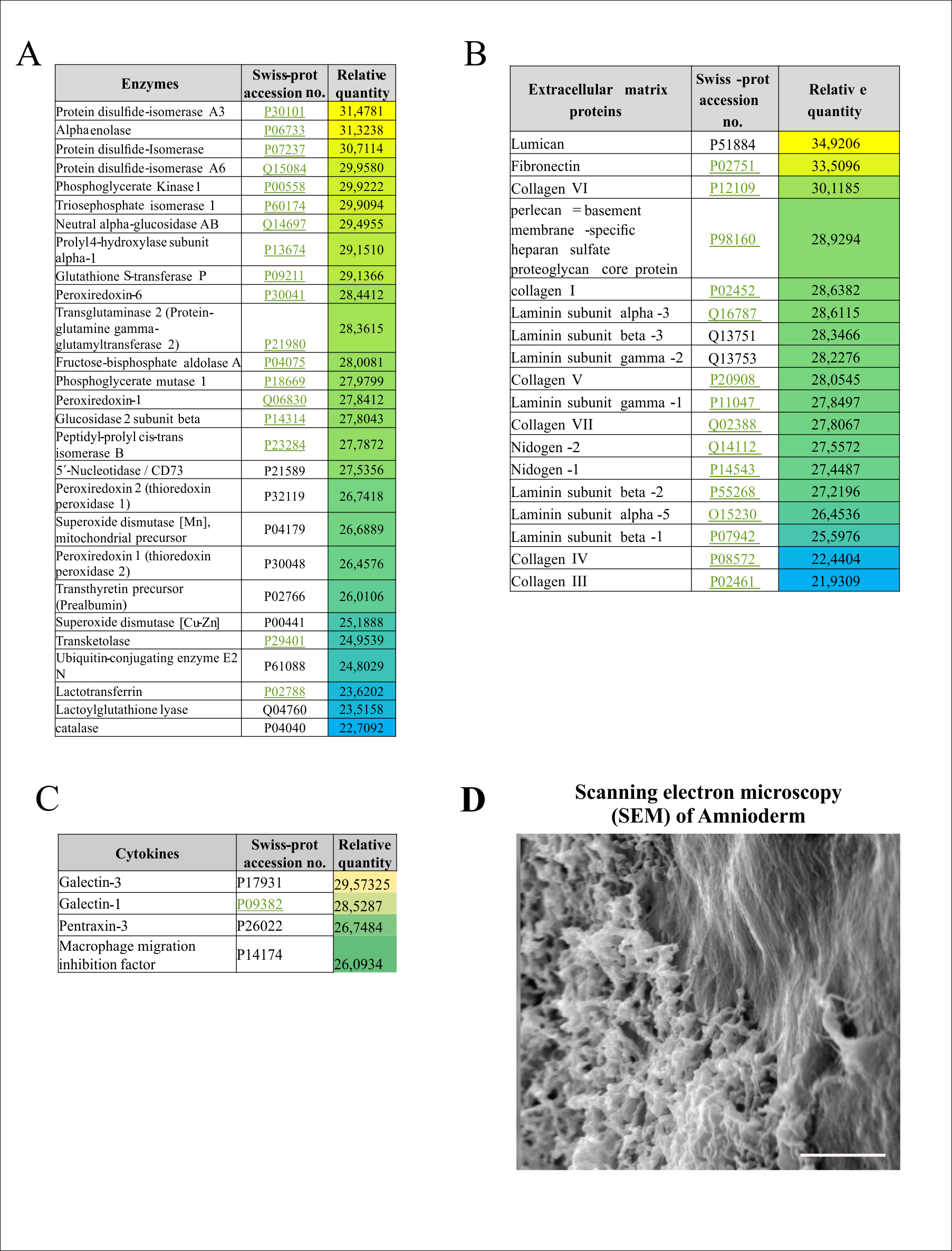

Supplement: Supplementary Figure 1 — Results of a liquid chromatography–mass spectrometry analysis of Amnioderm® revealed traces of 318 proteins. The proteins have been clustered into three major groups enrolled to wound healing: enzymes (A), extracellular matrix proteins (B), and cytokines (C). Relative quantities of the protein contents have been also color-coded within each group (yellow: highest content and blue: lowest); protein relative numbers below 20 have not been included in the analysis (cut-off). A microscopic structure of the Amnioderm® has been studied by scanning electron microscopy, which has revealed preservation of its native structure: basement membrane (smooth structure) and intermediate (spongy) layer. Scale bar = 2 μm. [file Image_1.TIF]
