# Supplementary material for: Case Report: Freeze-Dried Human Amniotic Membrane Allograft for the Treatment of Chronic Wounds: Results of a Multicentre Observational Study
Source: Front Bioeng Biotechnol. 2021 Jun 24;9:649446. doi: 10.3389/fbioe.2021.649446 (PMC8264202; doi:10.3389/fbioe.2021.649446)
Supplement: Supplementary Table 1 — Comparison of the Amnioderm therapy outcomes with similar studies using AM for chronic wound therapy. dHAM, dehydrated human amniotic membrane; dHACM, dehydrated human amniochorion membrane; NA, non-applicable. [file Table_1.docx]

|  | **Amnioderm** | **Epifix** | **NA** | **Epifix** | **NA** | **Epifix** |
| --- | --- | --- | --- | --- | --- | --- |
| **References** | current study | Zelen, 2014 | Mrugala, 2021 | Zelen, 2016 | Zelen, 2013 | Sheikh,2014 |
| **Type of the product** | dHAM | dHACM | dHACM | dHACM | dHAM | dHAM |
| **The median time of wound healing** | 8 weeks | 2,4 week | 64 days | 67,4 days | 4.2±3.1 weeks for the 10 patients healed. | 8 weeks |
| **Number of wounds in the study** | 16 | 40 | 6 | 32 | 11 | 4 |
| **Frequency of application** | weekly | weekly/biweekly | one application | weekly | weekly | both |
| **Type of wounds** | Diabetic /  non-diabetic chronic ulcers | Diabetic foot ulcers | Non-specified chronic wounds | Diabetic foot ulcers | Diabetic foot ulcers | Non-specified chronic wounds |
| **Percentage of the completely closed wounds** | 50% | 92,5% (12 weeks) | 4 responding wounds:  72% mean / 69% median decrease of wound size (3weeks) | 97% (12 weeks) | 91% (12 weeks) | Facilitated complete healing after 1-3 applications. |

# Supplement Table 1. Comparison of the Amnioderm therapy outcomes with similar studies using AM for chronic wounds therapy. Abbreviations: dHAM- dehydrated human amniotic membrane; dHACM -dehydrated human amniochorion membrane. NA – non-applicable
